# Performance of [^68^Ga]Ga-PSMA-11 PET/CT in patients with recurrent prostate cancer after prostatectomy—a multi-centre evaluation of 2533 patients

**DOI:** 10.1007/s00259-021-05189-3

**Published:** 2021-02-04

**Authors:** Ali Afshar-Oromieh, Marcelo Livorsi da Cunha, Jairo Wagner, Uwe Haberkorn, Nils Debus, Wolfgang Weber, Matthias Eiber, Tim Holland-Letz, Isabel Rauscher

**Affiliations:** 1 Department of Nuclear Medicine, Heidelberg University Hospital, Heidelberg, Germany; 2 Department of Nuclear Medicine, Inselspital, Bern University Hospital, University of Bern, Freiburgstr. 18, CH-3010 Bern, Switzerland; 3 Department of Nuclear Medicine, Hospital Israelita Albert Einstein, São Paulo, Brazil; 4 Clinical Cooperation Unit Nuclear Medicine, German Cancer Research Centre, Heidelberg, Germany; 5 Department of Nuclear Medicine, Technical University of Munich, Munich, Germany; 6 Department of Biostatistics, German Cancer Research Center, Heidelberg, Germany

**Keywords:** Prostate cancer, PET/CT, Positron emission tomography, PSMA, Prostate-specific membrane antigen

## Abstract

**Purpose:**

To evaluate the performance of [^68^Ga]Ga-PSMA-11 PET/CT in the diagnosis of recurrent prostate cancer (PC) after prostatectomy in a large multicentre cohort.

**Methods:**

The centres, which contributed to this study, were the departments of nuclear medicine of Heidelberg (Germany), Technical University of Munich (Germany) and Albert Einstein Hospital of São Paulo (Brazil). A total of 2533 patients who were scanned with [^68^Ga]Ga-PSMA-11 PET/CT at 1 h p.i. due to recurrent PC after prostatectomy were included in this retrospective analysis. Exclusion criteria were as follows: patients with untreated primary tumour, previous chemotherapy or Xofigo®; those previously treated with exclusively external beam radiation therapy or HIFU; those referred for PSMA-therapy; and those treated with ADT (including first- and second-generation ADT) within the last 6 months. Potential influences of different factors such as PSA level, PSA doubling-time (PSA_DT_), PSA velocity (PSA_Vel_), Gleason Score (GSC, including the separate analysis of 7a and 7b), age and amount of injected tracer were evaluated in a multivariable analysis.

**Results:**

The rate of pathologic PET/CT-scans was 43% for PSA ≤ 0.2 ng/ml, 58% for PSA > 0.2 to ≤ 0.5, 72% for PSA > 0.5 to ≤ 1.0 and increased to a maximum of 93% for PSA > 10 ng/ml. A pathological PET/CT was significantly (*p* = 0.001) associated with PSA level and higher GSC. Amount of injected tracer, age, PSA_DT_ and PSA_Vel_ were not associated with a higher probability of a pathological scan.

**Conclusion:**

[^68^Ga]Ga-PSMA-11 PET/CT at 1 h p.i. confirmed its high performance in the largest patient cohort yet analysed. Tumour detection showed a clear association with higher PSA and higher GSC. No association was found between a pathological [^68^Ga]Ga-PSMA-11 PET/CT and age, amount of injected tracer, PSA_DT_ or PSA_Vel_.

**Supplementary Information:**

The online version contains supplementary material available at 10.1007/s00259-021-05189-3.

## Introduction

The detection of recurrent prostate cancer (PC) with conventional imaging modalities such as bone scan (BS), computed tomography (CT) or magnetic resonance imaging (MRI) remains challenging. Within a remarkably short period following its first clinical introduction in May 2011 [1], positron emission tomography (PET) using [^68^Ga]Ga-PSMA-11 became the imaging modality of choice for the detection of recurrent PC wherever possible. [^68^Ga]Ga-PSMA-11 is a ^68^Ga-labelled small molecule inhibitor of the prostate-specific membrane antigen (PSMA), a transmembranous protein, which is significantly overexpressed in the majority of PC. Owing to its superior performance demonstrated in a multitude of studies, it has largely replaced previous generation tracers such as choline-PET/CT and shows high tumour contrast, sensitivity and excellent positive predictive value (PPV) of > 95% [2–5].

In keeping with the increasing publications and utilization of PSMA-PET/CT worldwide, there has been a rapid increase of knowledge about this diagnostic modality. The focus of some publications has been the interplay of possible factors that could predict PET positivity or tumour detection in patients with recurrent PC [2–4, 6–8]. One of the critical characteristics of the majority of these publications is the inhomogeneity in the patient cohorts, e.g. including patients with different initial therapies. Patients with initial exclusive external beam radiation therapy or high-intensity focused ultrasound (HIFU) of the primary tumours should not be mixed with prostatectomy patients since the prostate-specific antigen (PSA) values of both groups cannot be compared in the setting of biochemical relapse of PC. In addition, the inclusion of individuals with previous chemotherapy, Xofigo® or second-generation androgen deprivation therapy (ADT) is also critical since all these options indicate an advanced disease instead of a proper recurrence after initial therapy.

With regard to ADT, it was assumed for a long period that recurrent tumours in patients undergoing ADT had a higher probability of being detected when compared to patients without ADT [2–4]. However, in 2018 a study indicated that this effect is likely because ADT is usually prescribed in more advanced disease [9]. The same study found that uptake of [^68^Ga]Ga-PSMA-11 in castration sensitive PC is often significantly reduced by ADT. Interestingly, one third of patients with complete PSA response to long-term ADT still presented with a pathologic tracer uptake in tumour lesions. The reasons for the latter phenomenon are poorly understood. Considering all mentioned information, ADT can potentially make the results of the PSMA-PET/CT unpredictable [9]. Consequently, patients with an ongoing ADT should be excluded from studies that analyse the performance of PSMA ligands for the detection of early recurrent PC.

With all above-mentioned limitations in mind, there seems a more detailed data analysis possible. The aim of our study was to present more robust data regarding the performance of [^68^Ga]Ga-PSMA-11 PET/CT in a large patient cohort with recurrent PC.

## Materials and methods

### Patients

For this retrospective analysis, three different departments of nuclear medicine were requested to enter as many patients as possible to the study database. The three were the University Hospital of Heidelberg (Germany), the Technical University of Munich (Germany) and the Hospital Israelita Albert Einstein of São Paulo (Brazil). Patients’ characteristics are presented by Table 1.

**Table 1:** Patient characteristics. *SD* standard deviation

	Age (y) [*n* = 2533; missing: 0]	Tracer (MBq) [*n* = 2530; missing: *n* = 3]	GSC [*n* = 2112; missing: *n* = 421]	PSA at PET (ng/ml) [*n* = 2407; missing: *n* = 126]
Average	67	186	8	3.7
SD	8	53	1.1	25.5
Range	39–96	52–480	3–10	0.01–1055
Median	68	181	7	0.8
Prostatectomy *n* = 2533 (all patients)			Prostatectomy + radiation therapy *n* = 719	
PSA doubling time (months) [*n* = 558; missing: *n* = 1975]				
< 1 month (*n* = 13**)**	1- < 3 months (*n* = 64)	3- < 6 months (*n* = 122)	6- < 12 months (*n* = 98)	≥ 1 year (*n* = 261)
PSA velocity (ng/mL/y) [*n* = 324; missing: *n* = 2209]				
< 1 (*n* = 190)	1- < 3 (*n* = 65)	3- < 6 (*n* = 34)	6- < 12 (*n* = 17)	≥ 12 (*n* = 18)

#### Inclusion criteria

Patients referred for PSMA imaging due to recurrent PC after prostatectomy. Recurrent PC was defined as any PSA increase after prostatectomy. In patients with very low PSA closely above zero, recurrent PC was suspected by the referring physicians in alternative diagnostic tools since PSA was not a reliable parameter due to the aggressive characteristics of the tumour.

#### Exclusion criteria

Patients with untreated primary tumour, previous chemotherapy or Xofigo®, those previously treated with exclusively external beam radiation therapy or HIFU, those referred for PSMA-therapy and those treated with ADT (including first- and second-generation ADT) within the last 6 months.

Only the first scan available was included in this analysis; subsequent repeat scanning was not considered. The total number of included patients was *n* = 2533, including 765 patients from Heidelberg, 800 patients from Munich and 968 from Sao Paulo.

Approximately one third of the patients from Munich and Heidelberg have been published in various studies including studies addressing the same questions as the current analysis. However, as mentioned in the introduction chapter, those analyses did not represent a homogeneous cohort of patients in a proper context of recurrent disease after radical prostatectomy.

### Radiotracer

[^68^Ga]Ga-PSMA-11 was produced as previously described [10] and was applied to the patients via an intravenous bolus injection (mean of 186 ± 53 MBq, range 52–480 MBq). Variation of injected radiotracer activity was caused by the short physical half-life of ^68^Ga (68 min), variable elution efficiencies of the ^68^Ge/^68^Ga generator and unexpected delays in the scanning of patients.

### Imaging and image analysis

All patients were scanned according to the institutional protocols (details included in supplementary data) beginning at 1 h post-injection of the tracer with the CT scan (low-dose in Heidelberg and Sao Paulo, contrast enhanced in Munich) followed by the PET scan which was acquired in 3-D mode. The emission data were corrected for randoms, scatter and decay. Attenuation correction was performed using the CT data. Furosemide was not applied routinely in Heidelberg and Sao Paulo but was applied routinely in Munich. No hydration standards existed at all three institutes.

For the current analysis, the official clinical reports of the PET/CT were evaluated, which were created according to the institutional standards (details included in supplementary data).

### Statistical analysis

For statistical analysis, Excel 2010 (Microsoft, Redmond, USA) and R version 3.5.2 were used. In all cases a *p* value of < 0.05 was considered statistically significant. In addition to standard descriptive statistics, the following statistical analyses were performed:

1. Association between positive (pathologic) PET/CT results and the variables age, amount of injected tracer, PSA level and initial GSC was investigated jointly using a multivariable logistic regression model. The positive PET/CT result was defined as the dependent variable. Explanatory variables were defined as follows:

- Centre was included as a categorical variable (Heidelberg/Munich/São Paulo). Heidelberg was defined as the reference centre.
- GSC was included as a categorical variable and analysed divided in six different classes: GSC 5–6 (as reference; all other GSC classes were compared to GSC 5–6); GSC 7 (includes only patients without further specification); GSC 7a; GSC 7b; GSC 8 and GSC 9–10.
- Injected tracer amount was included as a continuous variable, expressed as multiples of 50 MBq. The odds ratio therefore always refers to a change by 50 MBq.
- Age was included as a continuous variable in multiples of 10 years.
- As PSA levels showed a skewed distribution, they were converted to a natural logarithmic scale (logPSA) first and included as a continuous variable. Odds ratios therefore refer to an increase by one log level.

The multivariable analysis was considered the primary analysis.

2) Association between positive (pathologic) PET/CT results and PSA_DT_ as well as PSA_Vel_ (both calculated using an official laboratory calculator from the LIMBACH group) was also investigated, using an extension of the multivariable logistic regression model from 1). As these variables were only available for a small fraction of our patients, both PSA_DT_ and PSA_Vel_ were investigated in two separate supplementary analyses based on a reduced collective for which all variables were available. In both cases, PSA_DT_ and PSA_Vel_ were coded as continuous variables. For the first analysis, only PSA_DT_ was added to the multivariable model from (1). For the second analysis, both PSA_DT_ and PSA_Vel_ were added to the model.

3) Proportions of positive PET results were calculated for subgroups defined by PSA levels and GSC classes. Exact binomial 95% confidence intervals were determined for these estimates.

## Results

**Fig. 1:**
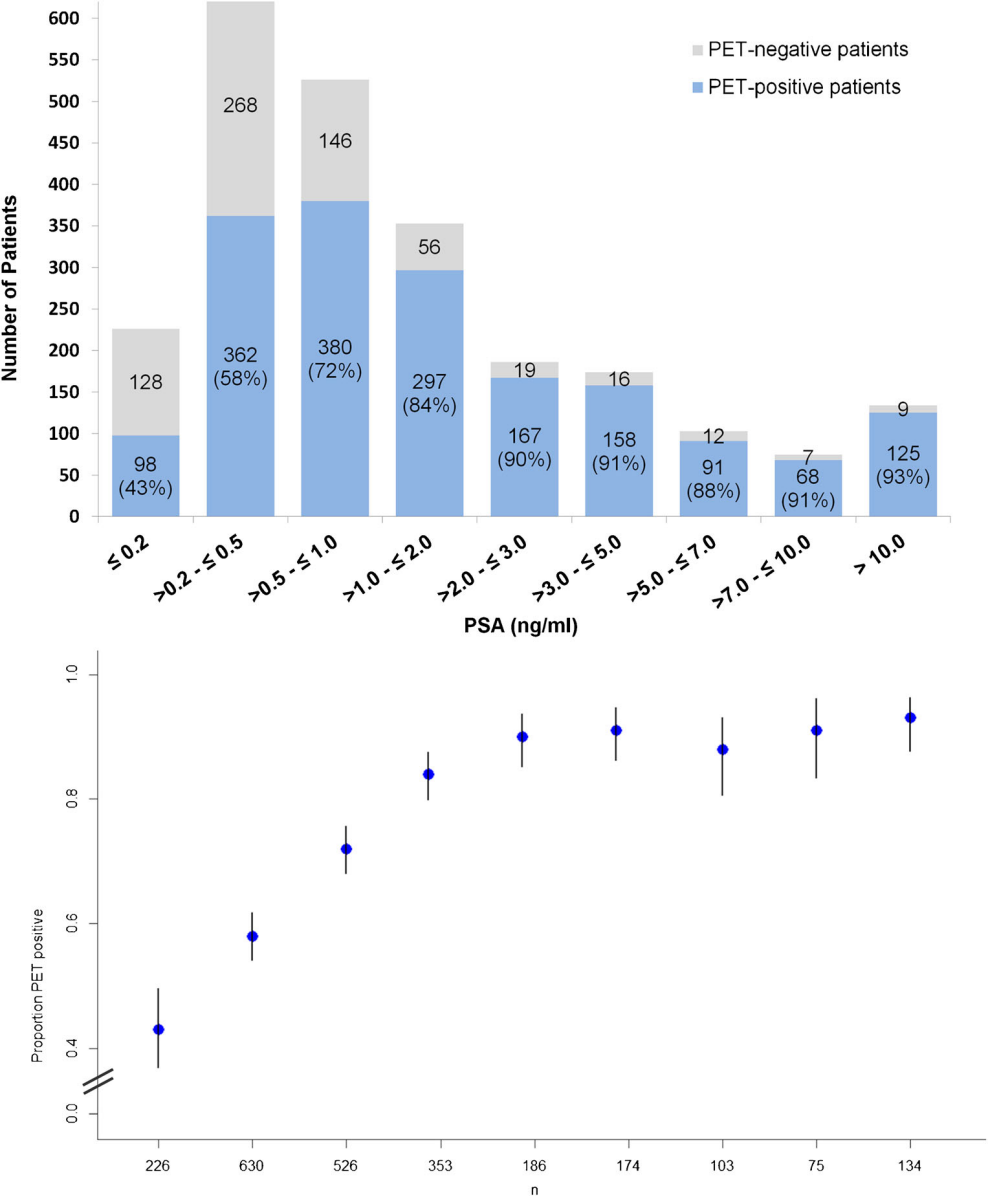
Probability of a pathologic [^68^Ga]Ga-PSMA-11 PET/CT as histogram and plot of the rates of pathologic scans with confidence intervals depending on PSA levels in 2407 patients. As shown by the figure, the probability of a pathologic scan rose with higher PSA values. The multivariable analysis demonstrated a significant association between a pathologic scan and higher PSA

In 1746 of 2533 patients (68.9%, = patient-based sensitivity), [^68^Ga]Ga-PSMA-11 PET/CT showed pathological PSMA-avid lesions indicative for PC. The patient-based sensitivities in different PSA subgroups are shown in Fig. 1. The probability of a pathologic scan started at 43% at PSA ≤ 0.2 ng/ml and rose with higher PSA values. Notably, the rates did not reach 100%, also not for the cohort of patients with the highest PSA (> 10 ng/ml).

**Fig. 2:**
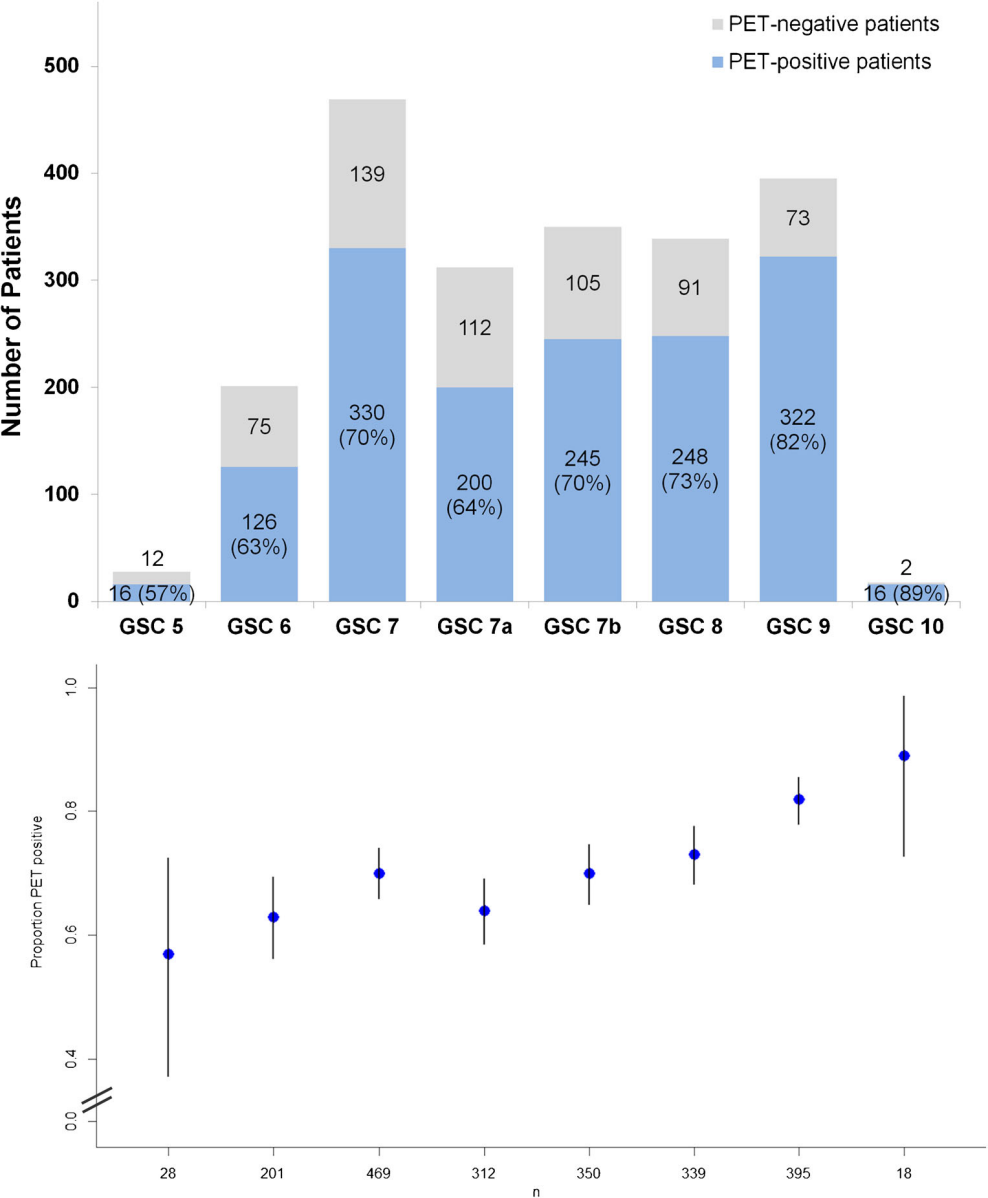
Probability of a pathologic [^68^Ga]Ga-PSMA-11 PET/CT as histogram and plot of the rates of pathologic scans with confidence intervals depending on GSC in 2112 patients. As shown by the figure, the probability of a pathologic scan rose with higher GSC, therefore with higher aggressiveness of the tumour. The multivariable analysis demonstrated a significant association between a pathologic scan and higher GSC

The probability of a pathologic [^68^Ga]Ga-PSMA-11 PET/CT depending on GSC is presented by Fig. 2. The probability of a pathologic scan increases with higher GSC, therefore with higher aggressiveness of the tumour.

A patient-based calculation of specificity, negative predictive value (NPV), and positive predictive value (PPV) was not applicable because no true-negative cases existed. All included patients were referred to PSMA-PET/CT due to recurrent PC.

Patients with pathologic radiotracer uptake (*n* = 1746) had a mean PSA of 4.65 ± 29.7 (0.01–1055, median of 1.09 ng/ml), a mean PSA_DT_ of 73.2 ± 257 months (0.1–4405, median of 9.7), a mean PSA_Vel_ of 3.3 ± 7.3 ng/mg/yr (0.02–51.8, median of 0.9) and a mean GSC of 7 ± 1.0 (range 3–10, median of 7) and were injected with a mean activity of 187 ± 53 MBq [^68^Ga]Ga-PSMA-11 (range 52–480 MBq, median 183 MBq).

Patients without pathologic radiotracer uptake (*n* = 787) had a mean PSA of 1.17 ± 5.3 (0.01–127, median of 0.4 ng/ml), a mean PSA_DT_ of 82.6 ± 156 months (0.4–964, median of 15.2), a mean PSA_Vel_ of 3.02 ± 19.8 ng/mg/yr (0.02–160.7, median of 0.7) and a mean GSC of 7 ± 0.9 (range 4–10, median of 7) and were injected with a mean activity of 184 ± 54.1 MBq [^68^Ga]Ga-PSMA-11 (range 66–400 MBq, median 178 MBq).

In the primary multivariable analysis based on the subset of *n* = 2043 patients where all analysed variables were available, a strong and significant association was detected between a positive PET result and both log PSA level and GSC. For every natural log step in PSA levels, the odds for a positive PET increased by a factor of 2.08. Even under the conservative estimate provided by the lower 95% confidence interval boundary for the odds ratio, an increase by a factor of 1.88 per natural log step can still GSC, a monotonous increase of pathologic scans was observed for increasing GSC with a maximum increase of odds by a factor of 3.53 being observed between the highest and lowest classes of GSC (i.e. GSC 9–10 vs. GSC 5–6). No significant or relevant association was detected between a positive PET result and the following parameters: PET centre (data not shown), age and amounts of injected radioactivity (Table 2, Model A).

**Table 2:** Potential influences of different factors on [^68^Ga]Ga-PSMA-11 PET/CT were evaluated by multivariable logistic regression analysis. GSC was classified as a categorical variable. (A) Main model with full sample size. (B) Model only including patients with available PSA doubling time (lnPSA_DT_). (C) Model only including patients with available lnPSA_DT_ and lnPSA_Vel_

A	Variables (*n* = 2043)	Coefficient	Standard error	95% Conf. lower limit	Odds ratio	95% Conf. upper limit	*P* value
	Munich	0.072	0.166	0.777	1.075	1.487	0.664
	Sao Paulo	0.250	0.154	0.950	1.284	1.737	0.014
	InPSA	0.732	0.052	1.876	2.080	2.305	< 0.001
	Age/10y	0.082	0.071	0.945	1.086	1.247	0.245
	Tracer/50 MBq	−0.012	0.061	0.877	0.988	1.112	0.838
	GSC 7	0.476	0.200	1.088	1.610	2.381	0.017
	GSC 7a	0.521	0.192	1.155	1.684	2.455	0.007
	GSC 7b	0.739	0.197	1.424	2.094	3.078	< 0.001
	GSC 8	0.745	0.203	1.417	2.107	3.134	< 0.001
	GSC 9 + 10	1.260	0.207	2.351	3.526	5.289	< 0.001
B	Variables (*n* = 463)	Coefficient	Standard error	95% Conf. lower limit	Odds ratio	95% Conf. upper limit	*P* value
	Munich	0.355	0.550	0.485	1.426	4.189	0.519
	Sao Paulo	0.548	0.557	0.580	1.729	5.156	0.326
	InPSA	0.727	0.119	1.640	2.070	2.612	< 0.001
	Age/10y	0.205	0.162	0.894	1.228	1.686	0.206
	Tracwe/50 MBq	0.033	0.129	0.803	1.034	1.331	0.795
	GSC 7	−0.080	0.479	0.361	0.923	2.361	0.868
	GSC 7a	0.598	0.405	0.822	1.818	4.022	0.140
	GSC 7b	−0.193	0.443	0.346	0.825	1.966	0.663
	GSC 8	0.167	0.438	0.501	1.182	2.787	0.702
	GSC 9 + 10	0.972	0.476	1.039	2.642	6.718	0.041
	InPSA-DT	−0.132	0.125	0.687	0.877	1.119	0.291
C	Variables (*n* = 269)	Coefficient	Standard error	95% Conf. lower limit	Odds ratio	95% Conf. upper limit	*P* value
	Munich	1.774	1.130	0.644	5.894	53.948	0.116
	Sao Paulo	2.225	1.217	0.852	9.257	100.530	0.067
	InPSA	0.540	0.253	1.046	1.716	2.815	0.033
	Age/10y	0.160	0.242	0.730	1.173	1.886	0.509
	Tracer/50 MBq	0.115	0.236	0.706	1.122	1.783	0.627
	GSC 7	−0.470	0.617	0.186	0.625	2.094	0.446
	GSC 7a	0.612	0.571	0.602	1.845	5.653	0.284
	GSC 7b	−0.452	0.590	0.200	0.637	2.023	0.444
	GSC 8	−0.085	0.645	0.260	0.919	3.251	0.895
	GSC 9 + 10	0.639	0.716	0.466	1.895	7.708	0.372
	InPSA-DT	0.096	0.263	0.658	1.101	1.842	0.714
	InPSA-Vel	0.429	0.265	0.912	1.535	2.583	0.106

In our separate analyses with the additional inclusion of PSA_DT_ (*n* = 463) and the analysis including both, PSA_DT_ and PSA_Vel_ (*n* = 269), we did not observe significant effects of either variable (Table 2, Model C). However, borderline significant effects were observed for PSA velocity, with a relevant but not significant odds ratio of 1.54 (*p* = 0.106).

## Discussion

The aim of our retrospective analysis was to present a more accurate evaluation of the performance of [^68^Ga]Ga-PSMA-11 PET/CT in a large multicentre patient cohort with recurrent PC after prostatectomy by considering recent knowledge about PSMA imaging. Only patients with recurrent PC after prostatectomy were included in this largest cohort yet analysed.

Among the patients included in this analysis, 68.9% presented with at least one lesion characteristic for PC in [^68^Ga]Ga-PSMA-11 PET/CT. This rate is lower compared with the second-largest and third-largest patient cohort including 1007 and 635 patients with overall PET positivity rates of 79.5 and 75%, respectively [4, 7]. This difference finds potential explanation in the lower median PSA of our cohort (0.8 ng/ml) compared with the two before-mentioned publications (2.2 ng/ml and 2.1 ng/ml, respectively).

The PET positivity rate was significantly associated with the PSA value beginning at 43% for PSA < 0.2 ng/ml and rising to 93% at PSA > 10 ng/ml (Fig. 1). A direct comparison of each PSA subgroup to previously published data is challenging because of different subgroup definitions. However, for PSA < 0.2 ng/ml, PET positivity rates of 46–47% have been reported [2, 4]. These rates are higher when compared with our analysis (43%). For PSA values up to 0.5 ng/ml, 38–58% PET positivity rates have been previously observed [3, 7]. These differences find explanation in the patient selection. As mentioned before, our exclusion criteria were stricter compared with previous studies thereby being closer to the “true rate” for this type of patients. In addition, we note that our data present with closer confidence intervals in the PSA subgroups (Fig. 1 and Table 2) compared with previous data [4], suggesting more precise estimations of the corresponding positivity rates.

As previously reported in different studies, also in our analysis, the rate of pathologic scans did not reach 100% because a proportion of PC does not sufficiently express PSMA. In some other cases, tumour lesions could potentially be obscured by excretion of [^68^Ga]Ga-PSMA-11 via the urinary tract.

Attention should be drawn to the fact that all PET positivity rates presented by this evaluation are only valid for scans conducted at 1 h p.i.. It is known from several publications including different PSMA ligands that scans at time points later than 1 h p.i. (e.g. 1.5 h or 3 h p.i.) show the majority of PC lesions with higher tracer uptake and contrast [1, 11–15], therefore resulting in higher numbers of detected PC lesions as well as a higher number of patients with a pathologic scan [16, 17]. Despite these data in favour of late scans, [^68^Ga]Ga-PSMA-11 PET/CT is routinely conducted at 1 h p.i. in the majority of centres, probably because of its first described clinical setup [1].

The multivariable analysis also demonstrated a significant association between a pathologic [^68^Ga]Ga-PSMA-11 PET/CT and higher GSC. In previous studies including bigger cohorts of patients with available GSC (*n* = 221–864), the association between GSC and a higher probability of a pathologic PET/CT was controversial [2–4]. With 2112 patients with available GSC information included in the current analysis (Table 1), we assume that our calculation is sufficiently powered. Also here, we note that our data present with closer confidence intervals in the GSC subgroups (Fig. 2 and Table 2) compared with previous data [4], suggesting more precise estimations of the corresponding positivity rates. The positive impact of higher GSC on PSMA-PET/CT is supported by preclinical studies in which a positive correlation between higher GSC and PSMA expression has been reported [18–20].

In accordance with previous publications including patient cohorts >*n* = 200 [2–4], we did not find an association between a pathologic scan and age or injected tracer doses. However, we note that the common doses of injected [^68^Ga]Ga-PSMA-11 (2–3 MBq/Kg body weight) are sufficient for imaging at 1 h p.i. With regard to the above-mentioned observation that later imaging seems superior, higher injected doses than 2–3 MBq/Kg body weight seem consequent.

**Fig. 3:**
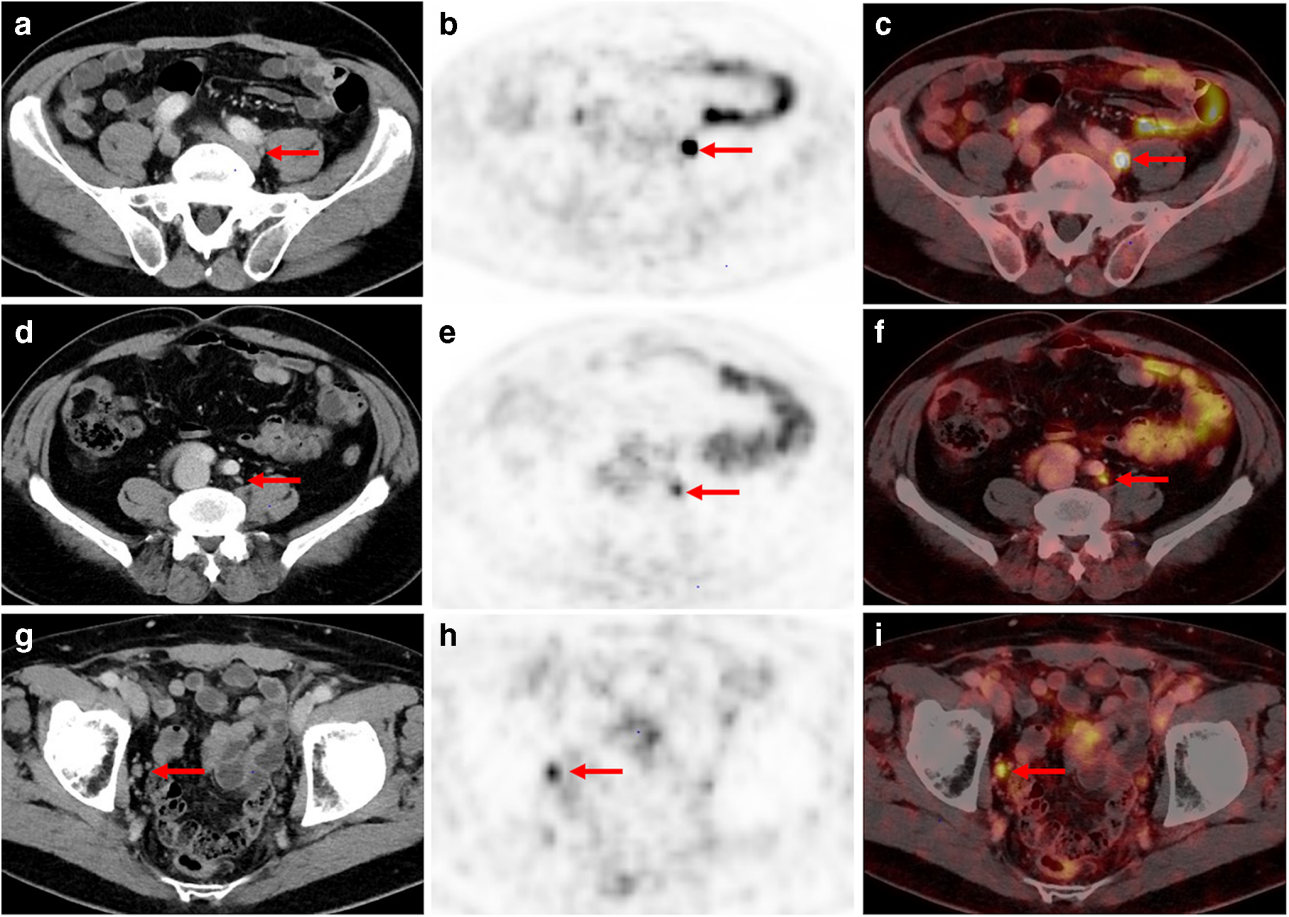
Examples of three different patients presenting with different tracer uptake intensities in histologically confirmed lymph node metastasis (LNM) despite comparable size of LN and clinical parameters. A-C: [^68^Ga]Ga-PSMA-11 PET/CT imaging of a 66-year old patient with recurrent PC (GSC 8; PSA level at PET examination 1.1 ng/ml). The patient presented with a correctly classified lymph node metastasis (LNM) behind the left common iliac artery with an intense, focal uptake on [^68^Ga]Ga-PSMA-11 PET (B, red arrow) and fused [^68^Ga]Ga-PSMA-11 PET/CT (C, red arrow). SUVmax of the LNM was 13.6. In the corresponding CT, only a small unsuspicious LN with a maximum diameter of 5 mm could be found (A, red arrow). D-F: 71-year old patient with PSA failure after radical prostatectomy (GSC 8; PSA level at PET examination 1.6 ng/ml) and a correctly classified LNM by [^68^Ga]Ga-PSMA-11 PET imaging: a morphologically completely unobtrusive lymph node is visible behind the left common iliac artery (axial diameter 5 mm) on sole CT imaging (A, red arrow) that shows intense, focal and thus suspicious tracer uptake on [^68^Ga]Ga-PSMA-11 PET (B, red arrow) and PET/CT fusion imaging (C, red arrow). SUV max of LNM was 4.1. G-I: 76-year old patient with biochemical recurrent PC (PSA value 0.77 mg/ml) after radical prostatectomy (GSC 9) presenting with a PSMA-positive LNM in the right obturator fossa (A and B, red arrow). Corresponding CT shows a slightly enlarged lymph node with an axial diameter of 10 mm (A, red arrow). SUV max of LNM was 5.1

In contrast to some studies including smaller patient cohorts [3, 8, 21, 22], our analyses showed no association between higher probabilities of a pathologic scan and PSA_DT_ or PSA_Vel,_ though the sample size for this analysis was still limited. Considering our data, a correlation between PSMA expression and tumour proliferation seems very unlikely. In fact, also our clinical experiences suggest that the contrast of PC lesions only depends on their PSMA expression: lesions of similar sizes and clinical constellations often present with different contrast—depending on their PSMA expression as shown by Fig. 3. We therefore assume that the statistical assessments of the mentioned studies including smaller patient cohorts were underpowered.

Beside the advantages of our current evaluation, namely, the more strict inclusion criteria, the huge cohort, the narrower confidence intervals and the multicentre character, we note several limitations of our study including the heterogeneity of imaging parameters such as diuretics administration. Recently, publications showed that the systematic administration of diuretics and hydration can help to increase tumour visibility [16, 23]. However, an early administration shortly after tracer injection could wash out the tracer before it had time to bind to PSMA, thereby reducing tumour visibility as well as image quality [23]. The optimal time of diuretics administration has yet to be defined. Our cohort also presents with many missing individual clinical data. For instance, we could not include an additional analysis of patients who had exclusively prostatectomy vs. those who had prostatectomy plus additional radiation therapy because data about the type of the radiation therapy (prostatic bed and/or lymphatic tract and/or concomitant temporary ADT) were insufficient.

Since 2018, it is known that ADT can make the results of PSMA imaging unpredictable. We therefore excluded patients who were treated with ADT within the last 6 months before the PET. Given the lack of evidence, it remains unclear whether 6 months free of ADT is sufficiently long for restoring the unaffected status of PSMA expression.

More limitations of our analysis are the lack of central standardized reading as well as the missing follow-up of patients in order to estimate the lesion-based negative and positive predictive values, commonly also referred to “specificity” of the methodology. One reason for the missing follow-up was that a substantial part of the patients was treated outside of our centres. Therefore, the access to reliable follow-up data was limited. However, with regard to biopsy- or histology-proven lesions, interpretation of retrospective data needs to be conducted with great caution because no standardized approach was followed: usually, mainly equivocal findings are further analysed by biopsy, thereby usually producing a bias. In addition, patients with multiple lymph nodes removed by surgery can also produce bias due to individual large number of tumour-affected lymph nodes. On the other hand, the excellent positive predictive value of [^68^Ga]Ga-PSMA-11 for recurrent PC has been demonstrated in a multitude of studies [2, 3, 7, 24–28]. According to these publications and our own experiences, any uptake of [^68^Ga]Ga-PSMA-11 above local background in morphologically visible lesions is highly specific for PC. Although PSMA ligand uptake has been reported for various benign and malignant tissues other than PC, their numbers are extremely low compared with those of PC lesions detected everyday by [^68^Ga]Ga-PSMA-11 PET/CT.

## Conclusion

In this multicentre analysis including the largest cohort yet analysed, [^68^Ga]Ga-PSMA-11 PET/CT at 1 h p.i. confirmed its overall high performance for the detection of recurrent PC. The probability of a pathologic scan is significantly associated with higher PSA levels and higher GSC. No association was found between higher probabilities of a pathologic [^68^Ga]Ga-PSMA-11 PET/CT and other factors such as age, injected amount of tracer, PSA_DT_ and PSA_Vel_.

## Supplementary Information

ESM 1 (DOCX 13 kb)
