# Supplementary material for: Performance of [68Ga]Ga-PSMA-11 PET/CT in patients with recurrent prostate cancer after prostatectomy—a multi-centre evaluation of 2533 patients
Source: Eur J Nucl Med Mol Imaging. 2021 Feb 4;48(9):2925–34. doi: 10.1007/s00259-021-05189-3 (PMC8263399; doi:10.1007/s00259-021-05189-3)
Supplement: Supplementary file 1 — (DOCX 13 kb) [file 259_2021_5189_MOESM1_ESM.docx]

**Supplementary data**

***Imaging Protocols***

*Heidelberg:* The patients of this evaluation were investigated with two different scanners. A Biograph-6 PET/CT scanner was used until August 2015 and was replaced by a Biograph mCT Flow scanner (both scanners made by Siemens, Erlangen, Germany). The two different PET/CT-scanners were cross-calibrated.

With regard to the Biograph-6 PET/CT, the scan protocol was as follows: A non-contrast-enhanced CT scan was performed 1 h post tracer injection using the following parameters: slice thickness of 5 mm, increment of 1.5 mm, soft tissue reconstruction kernel, 130 keV and 80 mAs. Immediately after CT scanning, a whole-body PET was acquired in 3-D (matrix 168×168). For each bed position (16.2 cm, overlapping scale 4.2 cm) a 4-min acquisition time with a 15.5-cm field of view (FOV) was used. The emission data were corrected for randoms, scatter and decay. Reconstruction was conducted with an ordered subset expectation maximization (OSEM) algorithm with 2 iterations/8 subsets and Gauss-filtered to a transaxial resolution of 5 mm at full-width at half-maximum (FWHM). Attenuation correction was performed using the low-dose non-enhanced CT data. Image analysis was performed using an appropriate workstation and software (Syngo TrueD, Siemens, Erlangen, Germany). No diuretics were applied between tracer injection and acquisition. Immediately prior to the scans, the patients were asked to empty the urinary bladder.

With regard to the Biograph mCT Flow scanner, a non-contrast-enhanced whole body CT scan was performed at 1h p.i. using the following parameters: slice thickness of 5 mm, increment of 3-4 mm, soft tissue reconstruction kernel, Care Dose. Immediately after CT scanning, a PET scan was acquired in 3-D (matrix 200x200) in flow motion with 0.7cm/min. The emission data were corrected for randoms, scatter and decay. Reconstruction was conducted with an ordered subset expectation maximization (OSEM) algorithm with 2 iterations/21 subsets and Gauss-filtered to a transaxial resolution of 5 mm at full-width at half-maximum (FWHM). Attenuation correction was performed using the low-dose non-enhanced CT data. PET and CT were performed using the same protocol for every patient on a Biograph mCT Flow scanner (Siemens, Erlangen, Germany). Image analysis was performed using an appropriate workstation and software (Syngo Via, Siemens, Erlangen, Germany).

No diuretics were applied between tracer injection and acquisition. Immediately prior to the scans, the patients were asked to empty the urinary bladder.

*Sao Paulo:* The patients were studied in two identical scanners, bought and installed at the same year: Biograph40 mCT (Siemens, Erlangen, Germany).

Scans were started 1h post-injection; data were acquired with time-of-flight (32-slice CT), with with 4 min per bed position. PET images were reconstructed using the Ultra-HD method (TrueX + time-of-flight) with 2 iterations, 21 subsets, 200×200 matrix and 3 mm Gaussian filter with non-enhanced CT-based attenuation correction (100 kV, 156 mAs, pitch 1.5, 0.5 s/rotation).

No diuretics were applied between tracer injection and acquisition. Before the beginning of the scan, patients were asked to void their bladder.

*Munich:* All patients underwent ^68^Ga-PSMA-11 PET/CT on a Biograph mCT scanner (4 ring TrueV model, 21.6 cm axial field of view; Siemens Medical Solutions, Erlangen, Germany). Scans were started 1h post-injection. PET/CT included a diagnostic CT (240 mAS, 120 kV, 5 mm slice thickness) in the portal venous phase (80 s after injection of an iodinated contrast agent (Imeron 300) followed by the PET scan. All patients received diluted oral contrast (300 mg Telebrix). Before the beginning of the PET/CT, patients were asked to void their bladder.

All PET scans were acquired in 3D-mode (matrix 200x200) with an acquisition time of 3-4 min per bed position. Emission data were corrected for randoms, dead time, scatter, and attenuation and reconstructed iteratively by an ordered-subsets expectation maximization algorithm (four iterations, eight subsets) followed by a post-reconstruction smoothing Gaussian filter (5-mm full width at half maximum). Image analysis was performed using an appropriate workstation and software (Syngo Via, Siemens, Erlangen, Germany).

***Image Analysis***

*Heidelberg:* All scans were first analyzed by three physicians at an interdisciplinary conference consisting of a board certified physician of nuclear medicine, an assistant physician of nuclear medicine and an assistant physician of radiology supported by a board certified physician of radiology. The reports were later controlled by the head of both departments or alternatively by their representatives (all board certified physicians). All available clinical data were considered by the reading physicians.

*Sao Paulo:* All scans were analyzed by two physicians: a board-certified physician of nuclear medicine (all with at least five years experience in PET/CT) and a board-certified physician radiologist, also experienced in reading PET/CT. The final reports were the result of the agreement of the findings. Divergences were solved by a consensus or consulting a third professional, another nuclear medicine physician or radiologist, whenever needed. All available clinical data were considered by the reading physicians.

*Munich:* All scans were first analyzed by three physicians at an interdisciplinary conference consisting of a board certified physician of nuclear medicine, an assistant physician of nuclear medicine and an assistant physician of radiology supported by a board certified physician of radiology. The reports were later controlled by the head of both departments or alternatively by their representatives (all board certified physicians). All available clinical data were considered by the reading physicians.
